# Towards Healthy Work Environments: Development and Validation of the Nursing Organizational Well-Being Questionnaire—A Theory-Based Measure

**DOI:** 10.3390/healthcare14101350

**Published:** 2026-05-14

**Authors:** Valerio Della Bella, Jacopo Fiorini, Alessandro Sili

**Affiliations:** 1 Nursing Department, Tor Vergata University Hospital, 00133 Rome, Italy; jacopo.fiorini@ptvonline.it (J.F.); alessandro.sili@ptvonline.it (A.S.); 2 JBI Italy Evidence-Based Practice and Health Research Center, 00136 Rome, Italy

**Keywords:** nurses, occupational health, organizational culture, working conditions, surveys and questionnaires, psychometrics

## Abstract

**Highlights:**

**What are the main findings?**

- The Nursing Organizational Well-being Questionnaire (NOW_Q) was developed as a theory-based, nursing-specific measure and demonstrated satisfactory psychometric properties, including validity and reliability.
- The NOW_Q identified eight core dimensions of nursing organizational well-being and distinguished three well-being profiles based on different configurations of nursing demands and resources, a novel capability compared with existing instruments.

**What are the implications of the main findings?**

- The NOW_Q offers nurse leaders and healthcare organizations a practical tool to assess organizational well-being in a more targeted and theory-informed way than currently available instruments.
- By identifying specific well-being profiles, the questionnaire can support tailored organizational interventions aimed at improving nurses’ well-being, strengthening work environments, and potentially enhancing care quality.

**Abstract:**

**Background/Objectives**: Nursing organizational well-being has important implications for nurses, patients, and healthcare organizations. From a nursing-specific perspective, it arises from the balance between nursing demands and nursing resources in the work environment. However, most available instruments are not grounded in explicit nursing theory and do not allow the identification of well-being profiles through person-centered approaches. This study aimed to develop and evaluate the psychometric properties of the Nursing Organizational Well-being Questionnaire (NOW_Q). **Methods**: Following COSMIN guidelines, a two-phase design was adopted. Phase 1 involved item generation and expert evaluation, resulting in a 28-item instrument rated on a 5-point frequency scale. Phase 2 consisted of a multicenter cross-sectional study. Construct validity was examined through exploratory and confirmatory factor analyses using cross-validation. Reliability was assessed using ordinal omega coefficients, concurrent validity through associations with a global organizational well-being item, and cluster analysis to explore practical utility. **Results:** Findings (*n* = 461 nurses; 7 hospitals) supported an eight-dimension structure: workload, emotional demands, work–family conflict, autonomy, available resources, nurse–nurse relationship, nurse–head nurse relationship, and nurse–physician relationship. The confirmatory model showed good fit (RMSEA = 0.051; CFI = 0.938; TLI = 0.927; SRMR = 0.067), and all dimensions demonstrated satisfactory internal consistency (ordinal omega = 0.75–0.87). Significant associations with global organizational well-being were observed. Three distinct profiles emerged (Nurturing, Observed-Detached, and Withstanding), reflecting different configurations of nursing demands and resources. **Conclusions**: The NOW_Q is a theory-based, nursing-specific instrument with satisfactory psychometric properties and practical utility for identifying organizational well-being profiles and supporting targeted interventions in clinical settings.

## 1. Introduction

### 1.1. Conceptual and Theoretical Foundations of Nursing Organizational Well-Being

Ensuring workers’ well-being should be a cornerstone of organizational strategies aimed at promoting employee satisfaction, motivation, and performance [1]. Within this broader scenario, nurses’ well-being has become a key priority for healthcare organizations [2]. However, the construct of well-being varies across occupations, as its definition and measurement depend on the theoretical model adopted [3]. Consequently, organizational well-being is often investigated in a limited way and assessed without fully capturing its complexity [4].

A recently developed Situation-Specific Theory defines nursing organizational well-being as “the result of nurses’ perceptions of their work environment, influenced by the organization’s ability to promote healthy working conditions. Positive interpersonal relationships within the professional team and the interaction between nursing work demands and the available nursing resources shape this well-being. By effectively addressing these demands and providing the necessary resources, organizations can enable nurses to achieve a state of well-being in their work and family lives, allowing them to provide high-quality patient care” [5].

According to this theory, nursing organizational well-being is not influenced by a single variable, but emerges from the balance between nursing demands—organizational characteristics requiring physical and psychological effort—and nursing resources, defined as work environment factors that support professional growth and goal achievement. Within this framework, organizational well-being is conceptualized as a dynamic condition shaped by how organizations balance these demands and resources within specific clinical contexts.

When an unfavourable imbalance between demands and resources occurs, nurses may experience negative health outcomes—including psychosomatic symptoms, stress, and burnout—with consequent repercussions on both patients and organizations [6]. Conversely, a harmonious balance between these dimensions is associated with better nurse health [7], higher quality of care [8] and more favorable organizational outcomes [9]. Nevertheless, these demands and resources may assume different relevance depending on the professional and the specific work context, potentially leading to different profiles of organizational well-being among nurses [10,11]. However, a clear theoretical operationalization of these dimensions is required to translate this framework into measurable constructs [5].

### 1.2. Theoretical Operationalization of Nursing Organizational Well-Being

According to the proposed Situation-Specific Theory of nursing organizational well-being [5], the development of the measurement instrument was grounded in a theory-driven operationalization of its core dimensions.

Consistent with broader theoretical perspectives emphasizing the interaction between job demands and job resources [7], nursing organizational well-being is structured around two overarching dimensions: nursing demands and nursing resources.

Nursing demands refer to work environment characteristics that require sustained physical, cognitive, or emotional effort. In the nursing context, these include workload, emotional demands related to patient and family interactions, and work–family conflict. These conditions have been consistently associated with adverse outcomes, including burnout, stress, and reduced quality of care [9,12].

Nursing resources encompass work environment characteristics that support professional functioning, facilitate goal achievement, and promote personal and professional development. These include professional autonomy, the availability of adequate resources, superior support, and effective relationships with nursing colleagues and other healthcare professionals. These factors are associated with higher job satisfaction, engagement, and positive organizational outcomes [7,13].

These domains provide the conceptual basis for specifying the dimensions of the construct, ensuring that the instrument reflects how nursing organizational well-being is theoretically generated through the balance between demands and resources.

### 1.3. Limitations of Existing Measures and Need for a Theory-Based Instrument

Exploring these variables and the instruments used to measure them remains complex for nursing researchers and managers [13,14]. The literature highlights substantial theoretical and methodological fragmentation, as existing questionnaires often capture only partial aspects of nursing organizational well-being, increasing the risk of measurement error and limiting the accurate estimation of relationships among variables [15]. Moreover, many questionnaires rely on non-nursing-specific frameworks, limiting their ability to account for the integrated role of nursing demands, resources, and interpersonal relationships [16].

Most available instruments focus on isolated dimensions of the work environment (e.g., Practice Environment Scale of the Nursing Work Index (PES-NWI), burnout measures, workload scales, or emotional job demand scales) and have been primarily validated using variable-centered approaches [3,17]. As a result, they provide limited insight into how different configurations of nursing demands and resources coexist within individuals and rarely allow the identification of distinct profiles of organizational well-being among nurses [10,11].

Furthermore, despite the availability of multiple instruments assessing the nursing work environment, no questionnaire has been explicitly developed to measure nursing organizational well-being within a coherent theoretical framework, nor grounded in the recently proposed Situation-Specific Theory [5]. This gap limits both the interpretability of findings and the development of evidence-based and tailored interventions [9,18]. In this context, the development of a theory-based instrument specifically designed for nursing settings becomes essential to capture the multidimensional and person-centered nature of organizational well-being [7,19].

### 1.4. Aim of the Study

For these reasons, the present study aims to evaluate the psychometric properties of a new questionnaire on nursing organizational well-being (NOW_Q), developed on the basis of a newly formulated situational theory. The availability of a psychometrically sound questionnaire may enable nurse managers and healthcare organizations to systematically assess organizational well-being and implement targeted interventions aimed at improving nurses’, patients’, and organizational health.

## 2. Materials and Methods

To conduct this study and develop and evaluate the NOW_Q, a two-phase approach was used, consistent with the COnsensus-based Standards for the selection of health Measurement INstruments. (COSMIN) [20]. The questionnaire was developed in Phase 1 and subsequently tested for psychometric validity in Phase 2.

### 2.1. Phase I—Questionnaire Development and Item Generation

In the first phase, a panel of eight experts with managerial and research experience in nursing was convened. The panel size is consistent with methodological recommendations suggesting that 6 to 10 experts are sufficient for content validity assessment [21,22]. Experts were purposively selected from both academic and clinical settings, including nurse managers and researchers, to ensure the integration of practical and theoretical perspectives in item evaluation. This multidisciplinary composition ensured both theoretical rigor and clinical relevance in the evaluation and refinement of the questionnaire items [21,22]. The experts met the following a priori eligibility criteria, adopted to ensure adequate expertise relevant to the construct under investigation: (a) doctoral-level education, to ensure familiarity with research methodology and theoretical frameworks; (b) demonstrated experience in nursing work environment or organizational well-being, defined as at least one of the following: (i) authorship of peer-reviewed publications in the field; (ii) teaching activities on related topics at undergraduate or postgraduate level; or (iii) current or previous managerial roles involving responsibility for nursing staff or organizational processes; and (c) more than five years of experience as registered nurses to ensure adequate exposure to organizational dynamics and clinical practice relevant to the construct under investigation.

The initial item pool was generated through three preliminary studies: (1) an integrative literature review aimed at identifying variables included in the main conceptual models of nursing organizational well-being [23], (2) a systematic review evaluating existing questionnaires used to measure nursing organizational well-being [16] and (3) a situation-specific theory of nursing organizational well-being that provided the conceptual foundation for the questionnaire [5]. Based on this evidence, a preliminary list of items was drafted and subsequently discussed during an expert panel meeting. Each expert independently evaluated every item using a dichotomous scale. Only items that reached unanimous agreement were retained, resulting in a preliminary pool of 38 items [22]. Consistent with the underlying theoretical framework, items were intentionally developed to capture both negatively (e.g., “During my work, I deal with demanding patients and family members”) and positively oriented work environment characteristics (e.g., “In my unit, everyone is committed to achieving the highest level of patient well-being”). When items overlapped with those used in existing questionnaires, permission to adapt them was obtained from the respective authors. The questionnaires consulted during item development included the PES-NWI [24]; the Work–Family Conflict Scale [25]; the Emotional Job Demand Scale [26]; the Nursing Organizational Health Questionnaire [27]; and the Quantitative Workload Inventory [28]. The expert panel agreed on the use of a five-point frequency Likert scale (1 = Never to 5 = Always) to ensure the measurement of observable work environment situations rather than subjective perceptions, in accordance with contemporary psychometric recommendations [29]. A five-point frequency scale was selected to capture the intensity of a unipolar construct, providing an appropriate balance between measurement precision, response variability, and respondent burden. Such formats are widely recommended in psychometric research and are suitable for factor-analytic approaches when distributional assumptions are reasonably met [21,29].

#### Face and Content Validity

Face and content validity were evaluated by a panel of 11 experts, including four nursing managers, three clinical nurses, two research experts, and two psychometricians.

Face validity was assessed through experts’ evaluation of item clarity and comprehensibility using a dichotomous response format (0 = no; 1 = yes). Experts were also invited to provide qualitative feedback to improve item wording and comprehensibility.

Content validity was evaluated by assessing the relevance and representativeness of each item with respect to the construct, using a 4-point Likert scale (0 = not relevant; 3 = highly relevant). A content validity index was calculated for each item (I-CVI) as the proportion of experts assigning a relevance score of 3 or 4 [21,22]. A questionnaire-level Content Validity Index (Q-CVI) was obtained by averaging all I-CVIs. Items and questionnaire relevance was deemed acceptable with an I-CVI and a Q-CVI, respectively, equal or more than 0.8 and 0.9 [21,22]. Items with I-CVI values between 0.70 and 0.79 were considered for revision, whereas items with I-CVI values below 0.70 were excluded [21,22]. The process of face and content validation led to the exclusion of ten items as their relevance was below 0.70, indicating that they were not acceptable [22]. Based on experts’ qualitative feedback, collected through open comments, items were reviewed and revised to improve clarity, wording consistency, and conceptual coherence (e.g., from “the nurse manager wants to be informed about work-related problems” to “In my unit, the nurse manager wants to be informed about work-related problems”). After removing the inadequate items, all remaining items achieved I-CVIs ≥ 0.82, and the overall Q-CVI was 0.91 (SD = 0.07), indicating excellent content validity. The final version of the NOW_Q used for psychometric testing consisted of 28 items. The preliminary item pool was designed to cover the core dimensions identified in the situation-specific theory, including nursing demands (emotional demands, workload, work–family conflict; e.g., “In my unit, I have too many things to do”) and nursing resources (Available Resources, Nurse–Nurse Relationship, Head Nurse Relationship, Nurse–Physician Relationship, and Autonomy; e.g., “In my unit, the nurse manager wants to be informed about work-related problems”). The results for face and content validity are reported in Supplementary Table S1.

### 2.2. Phase 2. Psychometric Testing

A multicenter cross-sectional study was conducted in Italy to evaluate the psychometric properties of the NOW_Q, including construct validity, reliability, criterion validity, and its practical utility in identifying organizational well-being profiles.

#### 2.2.1. Sampling

The study was conducted across seven Italian hospitals, predominantly public institutions, including both metropolitan and peripheral settings. All hospitals were acute care facilities covering a broad range of medical and surgical specialties, with variability in size and number of beds. A convenience sample of nurses who had been providing direct patient care for at least six months was recruited. Nurses were eligible regardless of shift type (day/night) or employment arrangement (full-time/part-time; temporary/permanent). Head nurses, nurse managers, and nurses with less than six months of experience in the clinical setting were excluded. Sample size adequacy followed psychometric guidelines recommending at least 10 participants per item [30,31]. Accordingly, a minimum sample of 380 participants was targeted based on the initial 38-item pool, which was subsequently reduced to 28 items following face and content validity assessment (see subsection “Face and Content Validity”).

#### 2.2.2. Data Collection

A web-based survey was distributed between January and June 2025 using institutional work email addresses. The first section provides information about the study’s purpose and includes a request for informed consent from participants. The second section consisted of the Nursing Organizational Well-being Questionnaire (NOW_Q). In addition, a single item assessing overall organizational well-being was administered to all participants. Nurses were asked to respond to the following question: “Thinking about your organization and the way you experience it, to what extent do you consider yourself to be in a state of organizational well-being?” Responses were provided on a Likert-type scale ranging from 0 to 10, with higher scores indicating higher perceived organizational well-being. The collected data were entered into an Excel worksheet, then extracted, coded, and compiled into an electronic dataset for statistical analysis.

#### 2.2.3. Ethics and Dissemination

The study respected the Helsinki Principles Declaration [32], and has been approved by the Ethics Committee of the University Hospital of Rome Tor Vergata (ethical approval number 172.24), with authorization obtained on 26 June 2024. All eligible nurses were informed about the study through an introductory page of the web-based survey, where informed consent was obtained prior to participation. Participants were also informed about data confidentiality and the possibility of withdrawing their consent at any time.

#### 2.2.4. Statistical Analysis

Statistical analyses were conducted in several stages. First, descriptive statistical analyses (mean M, standard deviation SD, range, absolute frequency N, and percentage F%, skewness, kurtosis) were calculated for all items and socio-demographic variables. Although the questionnaire items were measured on a 5-point Likert scale, they were treated as approximately continuous variables, consistent with psychometric literature supporting the use of Pearson correlations and maximum likelihood-based factor analytic approaches for ordinal variables with five or more response categories [33,34]. Construct validity was evaluated through exploratory factor analysis (EFA) and confirmatory factor analysis (CFA). Missing data were handled using listwise deletion for descriptive statistics and EFA, and Full Information Maximum Likelihood (FIML) for CFA [35]. A cross-validation approach was used, randomly splitting the sample into two sub-groups using the SPSS random split routine to select approximately 50% and 50% of study participants [36]. EFA was performed on subgroup A and CFA on subgroup B. Before conducting EFA, factorability was assessed through Bartlett’s test and the Kaiser–Meyer–Olkin (KMO) index. EFA was performed using the maximum likelihood extraction method. Factor retention was evaluated through eigenvalues (≥1), scree plot inspection, and parallel analysis for common factor analysis using O’Connor’s SPSS syntax [37]. Parallel analysis compares empirical eigenvalues with eigenvalues generated from randomly simulated datasets of the same size, retaining factors whose empirical eigenvalues exceed those expected by chance. Factors were subsequently rotated using Oblimin rotation to obtain an interpretable solution [38]. Item reduction was conducted using a combination of statistical and theoretical criteria, consistent with recommendations for scale development [29]. Specifically, items were evaluated based on: (1) communalities (h^2^ ≥ 0.30) [38], (2) factor loadings (≥|0.40| as an acceptable threshold) [39,40], (3) cross-loadings, defined as a difference of at least |0.20| between primary and secondary loadings [40], and (4) theoretical relevance [29].

Since the assumption of normality—assessed using the Shapiro–Wilk test [41]—could not be confirmed and given the ordinal nature of the 5-point Likert response format, CFA was conducted using the Maximum Likelihood estimator with robust standard errors (MLR), which is recommended for ordinal data treated as continuous [33,34]. The following fit indices were considered to evaluate the CFA model: Chi square (χ^2^) (not significant), RMSEA (<0.06), CFI (>0.90), TLI (>0.90) and SRMR (<0.08) [30,31]. The chi-square statistic (χ^2^) was reported but not used to judge model adequacy due to its sensitivity to sample size [42]. Consistent with current guidelines, the χ^2^/df ratio was not reported because its cutoffs are considered arbitrary; therefore, model evaluation relied on RMSEA, CFI, TLI, and SRMR [30]. Convergent validity was assessed using the average variance extracted (AVE), with a threshold of 0.50 considered indicative of adequate convergence [43]. Values below this threshold were evaluated in the context of the overall measurement model, taking into account composite reliability and discriminant validity [43,44]. Internal consistency reliability was determined using composite reliability (CR), ordinal-omega coefficients (ω) and Cronbach’s alpha (α), considered adequate if > of 0.70 [44,45]. Discriminant validity was assessed using the heterotrait–monotrait ratio of correlations (HTMT), computed in R (version 4.5.1) [46] using the lavaan and semTools packages. A conservative threshold of 0.85 was adopted, as recommended by Henseler et al. [47]. Factor loadings were considered adequate to confirm the construct if greater than |0.50| [30,31,40]. Loadings below this value were carefully evaluated and retained only when theoretically justified and when their inclusion did not adversely affect construct reliability or the overall measurement model [30,31,44]. Concurrent validity was assessed using Pearson’s correlations between the NOW_Q and a single global item measuring perceived organizational well-being [48,49]. The use of single-item global indicators as external criteria is supported by contemporary psychometric and occupational health research. While this approach does not capture the construct’s multidimensional structure and relies on a subjective appraisal, it provides a preliminary criterion-related validity [48,49]. Finally to explore the practical utility of the NOW_Q in identifying distinct organizational well-being profiles, a person-centered, cluster-based approach was applied [50] using a hybrid clustering strategy [44,51]. Prior to clustering, all variables were standardized (z-scores) to ensure comparability across scales. A hierarchical clustering using Ward’s method and squared Euclidean distances was first performed to inspect the dendrogram, agglomeration coefficients, and the interpretability of alternative cluster solutions. Solutions with different numbers of clusters were compared considering profile distinctiveness, conceptual coherence, and cluster sizes. The selected solution was then refined through K-means clustering. Cluster internal validity was evaluated with MANOVA on the NOW_Q dimensions, and external validity was assessed through ANOVA (Bonferroni post hoc) on a single-item criterion of perceived organizational well-being. A *p*-value < 0.05 was considered statistically significant. Descriptive statistics and cluster analysis were computed with SPSS v.25 [52]. The CFA was conducted with MPLUS v.8.5 [53] and JAMOVI (Version 2.6.44.0) was used [54,55] for ordinal-omega (ω) reliability coefficients.

## 3. Results

### 3.1. Participants’ Sociodemographic Characteristics

The study included 461 nurses (Table 1). Participants were predominantly female (73.97%). The mean age was 40.41 years (SD = 10.55). Most of the participants (F% = 54.44%) were married or in a civil union and held a Bachelor of Nursing degree (F% = 74.83%). Additionally, 55.10% reported having obtained a postgraduate qualification (Master’s degree). Participants had an average of 15.60 years of work experience (SD = 10.18), reporting a mean of 7.44 monthly overtime hours (SD = 9.76) and an average of 4.99 days of absence over the previous six months (SD = 9.76). Most participants worked in hospitals located in Central Italy (59.87%), followed by Northern (30.15%) and Southern Italy (9.98%). Nurses worked across multiple clinical settings. The most represented areas were medical departments (29.72%), followed by surgical units (17.57%). Finally, nurses reported caring for an average of 19.98 patients per shift (SD = 23.16), with an average staffing level of 4.99 nurses per shift (SD = 4.85).

### 3.2. Item Descriptive Statistics

The descriptive statistics of each item, including range, mean, SD, skewness and kurtosis are shown in Table 2. The means and the SD of items were often not excessively high or low. The majority of the items have a skewness and kurtosis indices within |1|. In addition, there was sufficient variance in the scoring.

### 3.3. Construct Validity

The validity and reliability of the NOW_Q were tested to ensure that the developed questionnaire was suitable to measure nurses’ organizational well-being.

#### 3.3.1. EFA in Sample A

The EFA was conducted on Sample A (*n* = 234). The KMO was 0.836, and Bartlett’s test was significant (χ^2^(378) = 3590.085, *p* < 0.001). To assess common method bias, Harman’s single-factor test was conducted using an unrotated exploratory factor analysis. The first factor accounted for 26.88% of the total variance, indicating that common method bias was not a major concern [56,57]. Maximum Likelihood extraction with Oblimin rotation was used, given the expected correlations among factors typically observed in occupational psychology [38,58]. Using the eigenvalue > 1 criterion [30] and scree plot inspection, eight factors were extracted (Emotional Demands, Work–Family Conflict, Workload, Available Resources, Nurse–Nurse Relationship, Nurse–Head Nurse Relationship, Nurse–Physician Relationship, Autonomy). Parallel analysis supported the presence of multiple factors exceeding chance levels, consistent with the multidimensional structure of the construct. These results were considered together with factor interpretability and theoretical coherence in determining the final factor solution. After rotation, these factors explained 65.21% of the total variance. All retained items met the predefined criteria for item adequacy, supporting the robustness of the final factor solution (Table 3).

#### 3.3.2. Final Structure of the NOW_Q

Factor 1 included the following 3 items: “In my unit, nurses and physicians have a good working relationship” (Item 22); “In my unit, there is collaboration between nurses and physicians” (Item 23); “In my unit, nurses and physicians share many work activities” (Item 24). This factor was named Nurse–Physician Relationship.

Factor 2 included the following 4 items: “In my unit, I have a say in how work activities are carried out” (Item 18); “In my unit, I can make decisions independently” (Item 19); “During my work, I can find solutions to problems that arise” (Item 21); “In my unit, I have the freedom to decide how to carry out my tasks” (Item 20). This factor was named Autonomy.

Factor 3 included the following 4 items: “In my unit, I have a lot of work to do” (Item 25); “In my unit, I am required to work very fast” (Item 27); “In my unit, I am required to work hard” (Item 26); “In my unit, I do not have enough time to complete my tasks” (Item 28). This factor was named Workload. Negative loadings for the Workload factor reflect the reverse semantic orientation of workload items [44].

Factor 4 included the following 3 items: “Work demands in my unit prevent me from completing the things I would like to do at home” (Item 6); “I feel so tired and stressed after work that it is difficult to fulfill family responsibilities” (Item 5); “Work commitments force me to change my family plans” (Item 7). This factor was named Work–Family Conflict.

Factor 5 included the following 3 items: “In my unit, the nurse manager wants to be informed about work-related problems” (Item 15); “In my unit, the nurse manager involves nurses in work-related decisions” (Item 17); “In my unit, the behavior of the nurse manager is consistent with stated goals” (Item 16). This factor was named Nurse–Head Nurse Relationship.

Factor 6 included the following 3 items: “Nurses in my unit are generally willing to meet organizational needs” (Item 9); “In my unit, everyone strives to achieve the best possible patient well-being” (Item 2); “In my unit, colleagues listen to each other and try to accommodate each other’s needs” (Item 11). This factor was named Nurse–Nurse Relationship.

Factor 7 included the following 3 items: “In my unit, there is sufficient staff to ensure the delivery of clinical activities” (Item 1); “In my unit, there are enough nurses to ensure high-quality patient care” (Item 3); “In my unit, there are adequate support services to allow sufficient time to be devoted to patients” (Item 4). This factor was named Available Resources.

Factor 8 included the following 3 items: “During my work, I deal with demanding patients and family members” (Item 12); “During my work, I deal with patients who complain continuously despite my efforts to help them” (Item 13); “During my work, patients and family members do not treat me with respect and courtesy” (Item 14). This factor was named Emotional Demands.

#### 3.3.3. CFA in Sample B

CFA was performed on the sub-group B (*n* = 227), testing the following models: one-factor model, two-factor model, and the eight-factor model. The one-factor CFA model (named Organizational Well-being) showed a poor fit to the data: χ^2^(350, *n* = 227) = 2296.72, *p* < 0.001; RMSEA = 0.157, 90% CI [0.150, 0.163], *p* (RMSEA < 0.05) < 0.001; CFI = 0.354; TLI = 0.302; SRMR = 0.141. The two-factor model also demonstrated inadequate fit: χ^2^(349, *n* = 227) = 1982.42, *p* < 0.001; RMSEA = 0.144, 90% CI [0.137, 0.150], *p* (RMSEA < 0.05) < 0.001; CFI = 0.458; TLI = 0.413; SRMR = 0.141. The eight-factor model showed an acceptable fit to the data, χ^2^(322, *n* = 227) = 548.324, *p* < 0.001; RMSEA = 0.056, 90% CI [0.048, 0.064], *p* (RMSEA < 0.05) = 0.121; CFI = 0.925; TLI = 0.912; SRMR = 0.070. After inspecting the modification indices, several theoretically justified residual correlations were added. Items 15 and 16 reflect highly related subcomponents of nurse leader support (communication openness and behavioural consistency); therefore, residual covariance was expected. According to Brown [30] and Kline [31], allowing residual covariance is appropriate when items share specific unique variance within a factor. Items 12 and 13 both assess emotionally demanding patient interactions. Literature on emotional demands [7,26] highlights that demanding and constantly complaining patients represent a single behavioural domain. According to Brown [30] and Kline [31], allowing correlated uniqueness is justified when items capture highly overlapping behavioural episodes. Items 1 and 3 use parallel wording assessing the same subfacet of staffing adequacy. According to Brown [30] and Kline [31], correlated uniqueness is warranted when two items share highly similar semantic content. The respecified model demonstrated good fit to the data: χ^2^(319, *n* = 227) = 505.847, *p* < 0.001; RMSEA = 0.051, 90% CI [0.042, 0.059], *p* (RMSEA < 0.05) = 0.429; CFI = 0.938; TLI = 0.927; SRMR = 0.067. All factor loadings were statistically significant and above |0.50| (Table 3). Only Item 12 (“During my work, I deal with demanding patients and family members”) showed a loading of |0.45|; however, it was retained due to its theoretical relevance, as interactions with patients and family members represent a core component of emotional job demands in nursing and are consistently identified in the literature as major sources of emotional workload [7,10,59]. Its retention therefore reflected a balance between preserving the theoretical coverage of the construct and adopting stricter psychometric criteria. Moreover, the item showed a limited impact on the overall reliability of the construct. The eight-factor CFA solution accounted for 61.5% of the total variance.

### 3.4. Convergent Validity and Reliability

Convergent validity was assessed using AVE. AVE values were 0.52 for *Emotional demands*, 0.62 for *Work–family conflict* and 0.62 for *Workload;* 0.63 for *Available Resources*, 0.47 for *Nurse–Nurse Relationship*, 0.67 for *Nurse–Head nurse relationship*, 0.68 for *Nurse–Physician Relationship* and 0.58 for *Autonomy*. All constructs exceeded the recommended threshold of 0.50, with the exception of *Nurse–Nurse Relationship* (AVE = 0.47), which fell slightly below the conventional criterion. However, this was considered acceptable given the satisfactory composite reliability and the adequate discriminant validity, as reported in the subsequent results and consistent with methodological recommendations [43]. CR values were 0.75 for *Emotional demands*, 0.83 for *Work–family conflict* and 0.87 for *Workload*; 0.84 for *Available Resources*, 0.81 for *Nurse–Nurse Relationship*, 0.85 for *Nurse–Head nurse relationship*, 0.86 for *Nurse–Physician Relationship* and 0.85 for *Autonomy*. Omega coefficient values were 0.75, 0.83, and 0.87 for *Emotional Demand*, *Work–Family Conflict*, and *Workload*, respectively; and 0.84, 0.81, 0.86, 0.86, and 0.85 for *Available Resource*, *Nurse–Nurse Relationship*, *Head Nurse Relationship*, *Nurse–Physician Relationship*, and *Autonomy*. Cronbach’s alpha was also calculated, with values ranging from 0.87 to 0.79 across dimensions. Coefficient reliability for the NOW_Q scale is reported in Table 3.

### 3.5. Discriminant Validity

The HTMT was computed to assess discriminant validity among the eight NOW_Q dimensions. All HTMT values were below the conservative threshold of 0.85, ranging from 0.08 to 0.68 (Table 4).

### 3.6. Concurrent Validity

The single item and the NOW_Q were administered concurrently to the entire sample. Pearson’s correlation coefficients were computed to evaluate the relationships between the organizational well-being item and each of the eight NOW_Q dimensions. Results showed significant correlations for all dimensions, with coefficients ranging from r = −0.36 to r = 0.37 (*p* < 0.001 for all associations) (Table 5).

### 3.7. Practical Validity

**Figure 1:**
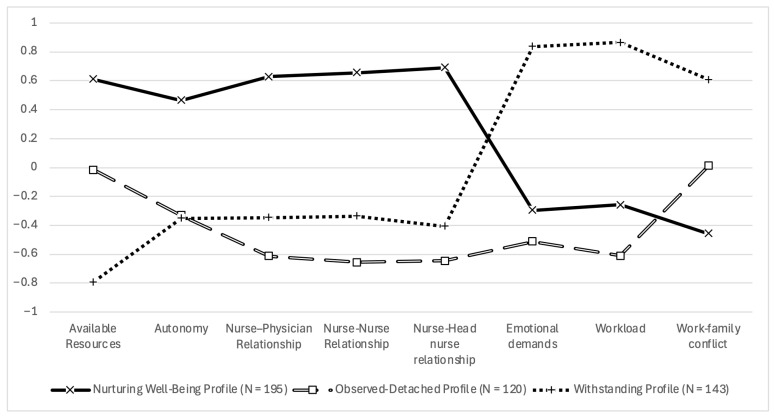
Standardized mean scores of the NOW_Q dimensions across the three well-being profiles.

To identify specific groups of nurses showing distinct profiles across the eight dimensions derived from the EFA and CFA, a cluster analysis was performed. Following the approach described by Barbaranelli [51], a hierarchical cluster analysis was first conducted using Ward’s method, with the eight CFA-derived dimensions (Emotional Demands, Work–Family Conflict, Workload, Available Resources, Nurse–Nurse Relationship, Nurse–Head Nurse Relationship, Nurse–Physician Relationship, and Autonomy) entered as grouping variables. The dendrogram and agglomeration schedule derived from the hierarchical clustering procedure were inspected to identify plausible cluster solutions and guide the selection of the number of clusters. The agglomeration coefficients suggested the presence of multiple distinct groups, with the largest increase observed in the final stages of cluster fusion, supporting the plausibility of solutions beyond a simple single or two cluster structure. Subsequently, K-means clustering was applied to compare and refine alternative cluster solutions. The two-cluster solution primarily reflected a broad distinction between more favorable and less favorable organizational well-being conditions, whereas the three-cluster solution produced balanced and clearly distinguishable groups (*n* = 195, *n* = 120, and *n* = 143) and provided a more differentiated and conceptually meaningful representation of nurses’ organizational well-being profiles across the eight dimensions. In contrast, the four-cluster solution resulted in greater fragmentation, including a smaller subgroup (*n* = 68) with partially overlapping characteristics and reduced conceptual distinctiveness. Therefore, the three-cluster solution was retained as the most parsimonious and interpretable representation of the data. Table 6 and Figure 1 present the profiles of the identified clusters across the eight dimensions.

A multivariate analysis of variance showed a significant multivariate effect of cluster membership, Wilks’ Lambda = 0.193, F(16, 896) = 71.41, *p* < 0.001, partial η^2^ = 0.560. Univariate ANOVAs, followed by Tukey–HSD post hoc tests, revealed that the three groups differed significantly on all factors, with the exception of Emotional Demands, where Clusters 1 and 2 did not differ, and Autonomy, where Clusters 2 and 3 showed no significant differences.

Cluster 1 (labelled *Nurturing Well-Being Profile*; *n* = 195, 42.58%) included nurses reporting high scores on relational dimensions (nurse–nurse, nurse–physician, nurse–head nurse relationships) and high levels of available resources, combined with low levels of work–family conflict, emotional demands, and workload.

Cluster 2 (labelled *Observed-Detached Profile*; *n* = 120, 26.20%) comprised nurses showing consistently low relational scores across all relationship dimensions, and lower emotional demands associated with patient and family interactions. Workload was perceived as low, available resources and work–family conflict were around the average, while autonomy was slightly below average.

Cluster 3 (labelled *Withstanding Profile*; *n* = 143, 31.22%) included nurses reporting very high workload, emotional demands, and work–family conflict, combined with low available resources and poor relational indicators across all relational domains.

Finally, a one-way ANOVA was conducted to compare the three clusters on the single-item measure of nursing organizational well-being used as an external criterion. The analysis showed a statistically significant difference between the three clusters in organizational well-being scores, F(2, 455) = 72.56, *p* < 0.001. Post hoc Tukey tests indicated that Cluster 1 reported the highest scores (M = 6.63, SD = 1.75), significantly higher than both Cluster 2 (M = 4.81, SD = 2.01; *p* < 0.001) and Cluster 3 (M = 4.25, SD = 1.99; *p* < 0.001). Cluster 2 also scored significantly higher than Cluster 3 (*p* < 0.05).

## 4. Discussion

The aim of this study was to develop and evaluate the psychometric properties of the NOW_Q, a theory-based questionnaire to assess nursing organizational well-being. The questionnaire had good psychometric properties and was able to capture, through its 8 dimensions, and 28 items, nursing-specific work environment variables that shaped organizational well-being, using a 5-point Likert scale. The NOW_Q addressed a longstanding gap by focusing on observable behavioral and environmental indicators rather than subjective evaluations. This methodological choice aligned with psychometric recommendations, which encouraged assessing observable workplace situations rather than agreement with generic statements, to reduce perceptual bias and improve measurement accuracy [57].

The interpretation of the findings was framed within the situation-specific theory of nursing organizational well-being, which conceptualized well-being as the result of the dynamic balance between nursing demands and nursing resources [5]. Content and face validity were supported by expert evaluations, with improvements in content validity indices across rounds, indicating adequate relevance, representativeness, and clarity of the items, and resulting in a refined 28-item questionnaire. The exploratory factor analysis identified an eight-factor structure consistent with the proposed theoretical framework [5]. Specifically, the situation-specific theory of nursing organizational well-being identifies three key variables within the domain of nursing demands: workload, emotional demands, and work–family conflict. Moreover, available resources, nurse–nurse relationships, nurse–head nurse relationships, nurse–physician relationships, and autonomy represented core variables within the domain of nursing resources [5]. These factors explained a substantial proportion of the total variance (65.21%), and all retained items met the predefined criteria for item adequacy, supporting the robustness of the factor solution. To evaluate the proposed structure, CFA was conducted on an independent subsample. The results indicated that the eight-factor model provided an acceptable fit to the data and a better fit than one-factor and two-factor models [30,31]. Comparison across the tested models supported the situation-specific theory of organizational well-being, which conceptualized well-being as a multidimensional construct [5]. Theoretical and empirical literature suggested that complex constructs could not be adequately represented through unidimensional models [15] and instead arise from the interaction of demands, resources, and social relationships [10].

Convergent validity was generally supported by the AVE results, with most dimensions exceeding the recommended threshold of 0.50. Only the AVE of the dimension *Nurse–Nurse Relationship* was slightly below this threshold (0.47); however, this was considered acceptable given the satisfactory composite reliability (0.81) and the overall coherence of the measurement model. This finding may have been explained by the multidimensional nature of collegial relationships in nursing, which encompassed different aspects of interpersonal interactions (e.g., collaboration, mutual support, and communication), potentially leading to greater heterogeneity among items [7,60]. Discriminant validity was supported by the HTMT results, with all values remaining below the conservative threshold of 0.85, indicating that the constructs were empirically distinct. Reliability estimates were satisfactory across all dimensions, indicating that the NOW_Q captured stable variations across key components of the work environment. Concurrent validity analyses showed significant and moderate correlations with a global measure of organizational well-being, consistent with the expectation that moderate correlations were appropriate when constructs were conceptually related but not redundant [61]. Moreover, recent studies reported modest associations between work demands and work resources, consistent with our findings [10]. One item (“During my work, I deal with demanding patients and family members”) showed a loading slightly below the recommended threshold (0.45); however, it was retained due to its theoretical relevance, as it captured a core aspect of emotional demands in nursing practice, and its inclusion did not adversely affect the overall measurement model [7,59].

The findings of this study where consistent with previous research highlighting the multidimensional nature of the nursing work environment and its association with organizational well-being [27,60]. In particular, the identification of both demand-related and resource-related dimensions was consistent with the proposed situation-specific theory and aligns with the current orientation of nursing research adopting the Job Demands–Resources framework [7,62]. However, unlike previous studies that often examined these components separately [8,14], the present findings supported a more integrated representation of the nursing work environment, in which demands, resources, and relational processes are simultaneously considered within a theoretically grounded, multidimensional measurement model.

Several widely used instruments also adopted a multidimensional approach to the assessment of the work environment; however, they typically conceptualized dimensions as relatively independent domains, might have led to a partial understanding of the organizational conditions that shape nurses’ well-being [19,63]. For example, the PES-NWI [24] focused on organizational and some relational aspects, whereas the Job Content Questionnaire (JCQ) primarily captured job demands and control dimensions, with limited attention to relational processes [17], The Copenhagen Psychosocial Questionnaire (COPSOQ) included a broad range of psychosocial factors across multiple dimensions [64]. Overall, these instruments remained highly heterogeneous, often lacking a unified theoretical grounding, containing large item pools, and failing to capture variables specific to nursing practice [19,63].

In contrast, the NOW_Q was developed within a situation-specific theoretical framework, and its structure reflected an integrated representation of the nursing work environment, in which demands, resources, and relational processes were conceptually and empirically interconnected.

Finally, the NOW_Q showed strong practical validity and its ability to identify distinct profiles of organizational well-being based on the measured dimensions. Two contrasting profiles emerged at the extremes: Nurturing and Withstanding.

In the *Nurturing* profile, nurses experienced a generally positive work environment characterized by adequate resources, functional professional relationships, greater autonomy, and manageable workloads [60]. From a Conservation of Resources Theory perspective, the availability of these “nursing resources” may activate resource gain spirals, reinforcing professional empowerment and adaptive coping processes, thereby contributing to higher levels of nursing organizational well-being [65]. In contrast, the *Withstanding* profile described nurses who were exposed to high demands and scarce resources, with heavy workloads, significant emotional demands, and more frequent work–family conflict; despite these conditions, these professionals continued to perform their role, striving to endure the strain [9]. According to Conservation of Resources theory, prolonged exposure to high demands combined with insufficient resources may progressively deplete personal and organizational resources, leading nurses to adopt a more endurance-oriented and defensive mode of functioning aimed primarily at maintaining role performance despite ongoing strain [65].

Between these extremes lay the *Observing–Detached* profile, which included nurses who appeared less engaged in tasks and professional relationships and for whom demands and resources do not act as either strong facilitators or barriers. This detachment was reflected in lower levels of organizational well-being [9]. From a Conservation of Resources theory perspective, this pattern may reflect a more passive or protective adaptation aimed at conserving remaining personal resources through reduced emotional and professional investment, which may partially explain the lower levels of organizational well-being observed in this group [65].

These profiles were consistent with the situation-specific theory and with person-centered approaches to occupational health, which emphasized heterogeneity in workers’ experiences [5,11]. Importantly, the profiles differed significantly in overall organizational well-being, demonstrating the questionnaire’s ability not only to measure discrete dimensions but also to provide actionable insights for managerial decision-making [19]. The NOW_Q may support targeted interventions tailored to the specific needs of each profile, reinforcing its relevance for clinical practice and organizational development.

### 4.1. Limits

The findings of this study should be interpreted in light of several limitations. First, the cross-sectional design introduced a potential selection bias, as the sample might not have fully represented the broader nursing workforce. Moreover, test–retest reliability and longitudinal validity were not explored, limiting conclusions about the questionnaire’s stability. Future longitudinal studies would have been needed to evaluate score stability and responsiveness. In addition, organizational well-being ratings might have been influenced by the socio-cultural characteristics of the participating settings. Although the multicenter design, including healthcare organizations from Northern, Central, and Southern Italy, helped mitigate the risk of geographic concentration, it could not have been entirely excluded. Moreover, the variability of organizational and regional contexts within the Italian healthcare system might have affected the findings, and the sample cannot be considered fully representative of the national nursing workforce. In addition, the study did not specifically account for variability across clinical units or departments within the participating organizations. Organizational well-being may differ substantially across micro-contexts characterized by distinct clinical demands, relational dynamics, coping strategies, and professional meanings. Previous qualitative evidence suggests that even highly demanding settings may include protective factors such as professional fulfillment, meaning-making, and intrinsic motivation [66]. Future studies should therefore explore the sensitivity of the NOW_Q across different clinical environments and assess potential unit-level variability through multilevel or stratified approaches. The sample size was determined based on psychometric recommendations related to the number of items; however, this approach did not ensure population representativeness, and information on the total eligible population was not available to estimate the proportion of participants included in the study. As the validation of the NOW_Q was conducted within the Italian context, future studies are needed to explore cross-country measurement invariance to confirm the generalizability of the results. A further limitation concerned the self-report nature of the questionnaire, which might have affected response accuracy. This risk was partially reduced by asking participants to report observable behaviors and situations rather than subjective judgments, yet perceptual bias might still have been present. Furthermore, concurrent validity was assessed using a single-item measure of organizational well-being, which might have limited the robustness of the findings and should be interpreted with caution, as it does not capture the multidimensional nature of the construct. Although the overall model fit was acceptable, the questionnaire structure showed room for refinement. Differences in the number of items across dimensions might have affected internal consistency, and some items—such as Item 12 within the Emotional Demands dimension—showed adequate but suboptimal loadings, reflecting a compromise between maintaining theoretical coverage of the construct and adopting stricter psychometric criteria. This suggests opportunities to improve item quality in future revisions. Finally, the cluster analysis was not externally validated, and the identified profiles should therefore be interpreted with caution. Further studies are needed to assess the stability and replicability of these profiles across different samples and settings. Despite these limitations, many were mitigated through the methodological rigor adopted, including a multi-stage validation process (EFA and CFA), the use of complementary analytical techniques, and the grounding of the questionnaire in a previously articulated situation-specific theory supported by empirical evidence. Collectively, these observations indicate the need to develop a more psychometrically balanced Version 2 of the questionnaire, with optimized factor structure, reduced self-report bias, and more homogeneous item distribution.

### 4.2. Implications for Practice, Future Research, and Management

The NOW_Q offers nursing leaders a practical questionnaire to rapidly identify organizational well-being profiles within clinical units, enabling targeted rather than generic interventions. It may also serve as a periodic screening questionnaire to monitor trends in organizational well-being and to evaluate the impact of managerial or organizational initiatives.

Future studies should focus on refining the questionnaire in a second Version, assessing its longitudinal stability, and examining its predictive validity in relation to organizational outcomes (e.g., absenteeism, performance) and patient care indicators. Cross-cultural and subgroup analyses (e.g., early vs. late career nurses, diverse clinical contexts) are also required to determine the generalizability of the theoretical framework and the robustness of the questionnaire across nations and settings.

## 5. Conclusions

This study developed and evaluated the NOW_Q, a theory-based questionnaire designed to assess nursing organizational well-being. The findings provided initial evidence supporting its multidimensional structure and psychometric adequacy. The instrument also showed potential in identifying distinct organizational well-being profiles, suggesting its possible applicability in supporting targeted interventions in clinical settings. Further research is needed to confirm these findings and refine the instrument. Nurses care for the well-being of their patients every day. These findings further support the importance of recognizing and promoting nursing organizational well-being as a relevant priority for healthcare organizations and clinical practice.

## Figures and Tables

**Table 1:** Socio demographic characteristics (*n* = 461).

	*n* (%)	Mean (SD)	Range
Nurse characteristics			
Age		40.41 (10.55)	20–64
Gender			
Male	109 (23.64)		
Female	341 (73.97)		
Non-binary	11 (2.39)		
Civil Status			
Single	171 (37.09)		
Separated/Divorced	37 (8.03)		
Married	251 (54.44)		
Widow/Widower	2 (0.43)		
Masters in science in nursing			
Yes	254 (55.10)		
No	207 (44.90)		
Working years		15.60 (10.18)	0–41
Monthly overtime hours for week		7.44 (9.76)	0–73
Last six months days of absence		4.99 (9.76)	0–90
Hospital and Ward Characteristics			
Institutional Macro Area			
North	139 (30.15)		
Center	276 (59.87)		
South	46 (9.98)		
Working Clinical Settings			
Medicine department	137 (29.72)		
Surgical department	81 (17.57)		
Emergency department	56 (12.14)		
Operatory theater	24 (5.21)		
Oncological department	11 (2.39)		
Outpatient clinic	39 (8.46)		
Intensive care unit	69 (14.97)		
Pediatric department	9 (1.95)		
Territorial care	32 (6.94)		
Nr of Nurse in the last shift		4.99 (4.85)	1–12
Nr of inpatients in the last shift		19.98 (23.16)	0–170

Note. *n* = absolute frequency; % relative frequency; SD = standard deviation.

**Table 2:** Item descriptive statistics (*n* = 461).

	Mean	SD	Skewness	Kurtosis
Item 1	3.27	1.10	−0.48	−0.51
Item 2	3.65	1.00	−0.59	0.05
Item 3	3.17	1.13	−0.38	−0.63
Item 4	2.88	1.11	−0.12	−0.85
Item 5	3.05	0.86	−0.25	0.05
Item 6	2.91	0.93	−0.19	−0.36
Item 7	3.09	0.94	−0.36	−0.17
Item 8	4.05	0.92	−0.80	0.07
Item 9	3.61	0.82	−0.64	0.48
Item 10	2.94	1.00	0.02	−0.49
Item 11	3.43	0.99	−0.50	−0.27
Item 12	3.69	0.97	−0.73	0.41
Item 13	3.23	0.99	−0.10	−0.52
Item 14	2.78	0.99	−0.03	−0.38
Item 15	3.86	1.15	−0.77	−0.34
Item 16	3.40	1.17	−0.45	−0.72
Item 17	3.24	1.26	−0.25	−0.94
Item 18	3.51	0.87	−0.55	0.40
Item 19	3.40	0.89	−0.44	0.24
Item 20	3.65	0.91	−0.70	0.41
Item 21	3.79	0.78	−0.61	0.78
Item 22	3.58	0.85	−0.60	0.29
Item 23	3.55	0.90	−0.49	0.07
Item 24	3.56	0.97	−0.54	−0.06
Item 25	4.08	0.76	−0.71	0.88
Item 26	3.88	0.84	−0.38	−0.01
Item 27	3.86	0.87	−0.36	−0.40
Item 28	3.09	0.96	0.08	−0.34

Note. Response ranged from 1 (Never) to 5 (Always) SD = standard deviation.

**Table 3:** Standardized Factor Loadings from the EFA (*n* = 234) and CFA (*n* = 227).

Item	Dimension															
	Nurse–Physician Relationship		Autonomy		Workload		Nurse–Head Nurse Relationship		Work–Family Conflict		Emotional Demands		Available Resources		Nurse–Nurse Relationship	
	EFA	CFA	EFA	CFA	EFA	CFA	EFA	CFA	EFA	CFA	EFA	CFA	EFA	CFA	EFA	CFA
Items 1													0.802	0.735		
Items 2															0.527	0.575
Items 3													0.944	0.831		
Items 4													0.611	0.820		
Items 5									0.710	0.826						
Items 6									0.893	0.854						
Items 7									0.801	0.675						
Items 8															0.450	0.577
Items 9															0.667	0.700
Items 10															0.448	0.724
Items 11															0.685	0.843
Items 12											0.769	0.450				
Items 13											0.834	0.738				
Items 14											0.624	0.895				
Items 15							0.718	0.590								
Items 16							0.940	0.845								
Items 17							0.749	0.976								
Items 18					−0.654	0.812										
Items 19					−0.844	0.884										
Items 20					−0.869	0.752										
Items 21					−0.761	0.662										
Items 22	0.939	0.828														
Items 23	0.870	0.959														
Items 24	0.654	0.657														
Items 25			0.743	0.816												
Items 26			0.834	0.889												
Items 27			0.799	0.757												
Items 28			0.608	0.669												
ω/α	0.86/0.84		0.85/0.84		0.87/0.85		0.86/0.87		0.83/0.82		0.75/0.79		0.84/0.87		0.81/0.80	

Note. The table reports the standardized factor loadings obtained from the exploratory factor analysis (EFA) and the confirmatory factor analysis (CFA). Values ≥ 0.50 are generally considered preferable, although lower values were retained when theoretically justified. ω = ordinal omega coefficients. α = Cronbach’s alpha.

**Table 4:** Heterotrait–Monotrait Ratio (HTMT) Matrix (*n* = 227).

	1	2	3	4	5	6	7	8
1. Nurse–Physician Relationship	-							
2. Autonomy	0.53	-						
3. Workload	0.08	0.11	-					
4. Nurse–Head nurse relationship	0.32	0.36	0.12	-				
5. Work–family conflict	0.27	0.36	0.40	0.37	-			
6. Emotional demands	0.15	0.16	0.32	0.20	0.33	**-**		
7. Available Resources	0.39	0.34	0.46	0.32	0.45	0.36	-	
8. Nurse–Nurse Relationship	0.47	0.38	0.13	0.68	0.34	0.16	0.38	-

**Table 5:** Pearson’s correlation between dimension and organizational well-being single items (*n* = 461).

	1	2	3	4	5	6	7	8	9
1. Nurse–Physician Relationship	-								
2. Autonomy	0.44 **	-							
3. Workload	−0.04	−0.14 *	-						
4. Nurse–Head nurse relationship	0.33 **	0.36 **	−0.13 *	-					
5. Work-family conflict	−0.17 *	−0.21 **	0.35 **	−0.24 **	-				
6. Emotional demands	−0.13 *	−0.13 *	0.32 **	−0.10 *	0.29 **	-			
7. Available Resources	0.35 **	0.24 **	−0.38 **	0.29 **	−0.30 **	−0.31 **	-		
8. Nurse–Nurse Relationship	0.43 **	0.33 **	−0.08	0.54 **	−0.24 **	−0.13 *	0.38 **	-	
9. Organizational Well-Being	0.32 **	0.36 **	−0.17 **	0.44 **	−0.36 **	−0.29 **	0.37 **	0.37 **	-

Note. *p* < 0.05 *; *p* < 0.001 **.

**Table 6:** Mean scores and (standard deviation) of the NOW_Q dimensions across the three well-being profiles.

Cluster	Available Resource	Autonomy	Nurse–Physician Relation	Nurse–Nurse Relationship	Nurse–Head Nurse Relationship	Emotional Demands	Workload	Work–Family Conflict	OW
Nurturing	3.71 (0.71) ^a^	3.92 (0.55) ^a^	4.00 (0.47) ^a^	4.07 (0.53) ^a^	4.23 (0.65) ^a^	2.98 (0.68) ^a^	3.54 (0.58) ^a^	2.66 (0.73) ^a^	6.63 (1.75) ^a^
Well-Being									
(*n* = 195)									
Observed	3.09 (0.83) ^b^	3.35 (0.73) ^b^	3.08 (0.60) ^b^	3.07 (0.78) ^b^	2.81 (0.94) ^b^	2.81 (0.79) ^a^	3.29 (0.67) ^b^	3.03 (0.69) ^b^	4.81 (2.01) ^b^
Detached									
(*n* = 120)									
Withstanding	2.32 (0.87) ^c^	3.33 (0.75) ^b^	3.30 (0.67) ^c^	3.28 (0.76) ^c^	3.07 (1.00) ^c^	3.92 (0.57) ^b^	4.35 (0.49) ^c^	3.50 (0.68) ^c^	4.25 (1.99) ^c^
(*n* = 143)									

Note. Different superscript letters indicate statistically significant differences between clusters at post hoc Tukey tests (at least *p* < 0.05). The overall effect of cluster membership was significant at *p* < 0.001 for both MANOVA and univariate ANOVAs. OW = Organizational well-being.

## Data Availability

The original data presented in the study are openly available in Zenodo at https://doi.org/10.5281/zenodo.19046129.

## Supplementary Materials

The following supporting information can be downloaded at: https://www.mdpi.com/article/10.3390/healthcare14101350/s1, Table S1: Results of Content Validity Across Two Expert Rounds (Round 1: *n* = 11; Round 2: *n* = 11).

## Abbreviations

The following abbreviations are used in this manuscript:

NOW_Q	Nursing Organizational Well-being Questionnaire
COSMIN	Consensus-based Standards for the selection of health Measurement INstruments
EFA	Exploratory Factor Analysis
CFA	Confirmatory Factor Analysis
FIML	Full Information Maximum Likelihood
MLR	Maximum Likelihood Robust estimator
KMO	Kaiser–Meyer–Olkin index
RMSEA	Root Mean Square Error of Approximation
CFI	Comparative Fit Index
TLI	Tucker–Lewis Index
SRMR	Standardized Root Mean Square Residual
MANOVA	Multivariate Analysis of Variance
ANOVA	Analysis of Variance
SD	Standard Deviation
N	absolute frequence
M	Mean
I-CVI	Item-level Content Validity Index
Q-CVI	Questionnaire-level Content Validity Index
CR	Composite Reliability
AVE	Average Variance Extracted
HTMT	Heterotrait–Monotrait Ratio of Correlations
α	Cronbach’s alpha
ω	Ordinal Omega reliability coefficient
JD-R	Job Demands–Resources model
PES-NWI	Practice Environment Scale of the Nursing Work Index
COPSOQ	Copenhagen Psychosocial Questionnaire
JCQ	Job Content Questionnaire
SPSS	Statistical Package for the Social Sciences
