# Supplementary material for: Towards Healthy Work Environments: Development and Validation of the Nursing Organizational Well-Being Questionnaire—A Theory-Based Measure
Source: Healthcare (Basel). 2026 May 14;14(10):1350. doi: 10.3390/healthcare14101350 (PMC13206091; doi:10.3390/healthcare14101350)
Supplement: Supplementary file 1 [file healthcare-14-01350-s001.zip › healthcare-4300224-supplementary.pdf]

**Table S1. Results of Content Validity Across Two Expert Rounds (Round 1: N = 11; Round 2: N = 11)**

| Round 1 | | | Round 2 | | |
|---|---|---|---|---|---|
| **#items** | **Statement** | **I-CVI** | **Statement** | **I-CVI** | **#items** |
| 1 | There is a lot of work to do | .73 | In my unit, I have a lot of work to do | .91 | 25 |
| 2 | Nurses and physicians have a good working relationship | .73 | In my unit, nurses and physicians have a good working relationship | .91 | 22 |
| 3 | Excessive workload prevents me from completing the things I would like to do at home | .82 | Work demands in my unit prevent me from completing the things I would like to do at home | .91 | 6 |
| 4 | I have a say in how activities are carried out | .91 | In my unit, I have a say in how work activities are carried out | .91 | 18 |
| 5 | The nurse manager wants to be informed about work-related problems | 1.00 | In my unit, the nurse manager wants to be informed about work-related problems | 1.00 | 15 |
| 6 | Each nurse's work represents a significant contribution | 1.00 | In my unit, each nurse's work represents a significant contribution to clinical activities | 1.00 | 8 |
| 7 | The work is emotionally demanding | .64 | ***Removed*** | | |
| 8 | There are aspects of the work that affect me personally | .55 | ***Removed*** | | |
| 9 | I can make decisions independently | 1.00 | In my unit, I can make decisions independently | 1.00 | 19 |
| 10 | My work activities are influenced by the demands of my family (or partner) | .64 | ***Removed*** | | |
| 11 | I deal with emotionally demanding situations | .64 | ***Removed*** | | |
| 12 | The tasks I have to perform require knowledge and skills that I do not possess | .55 | ***Removed*** | | |
| 13 | Nurses are generally willing to meet organizational needs | .82 | Nurses in my unit are generally willing to meet organizational needs | .82 | 9 |
| 14 | In the team, everyone strives to achieve the best possible outcomes (patient well-being) | 1.00 | In my unit, everyone strives to achieve the best possible patient well-being | 1.00 | 2 |
| 15 | There is collaboration between nurses and physicians | .91 | In my unit, there is collaboration between nurses and physicians | .91 | 23 |
| 16 | I deal with users who do not treat me with due respect and courtesy | .91 | During my work, patients and family members do not treat me with respect and courtesy | .91 | 14 |
| 17 | I have to sacrifice work in order to spend more time at home | .55 | ***Removed*** | | |
| 18 | I have to find solutions to the problems that arise | .82 | During my work, I can find solutions to problems that arise | 1.00 | 21 |
| 19 | There is sufficient time and opportunity to discuss care-related issues among nurses | .73 | In my unit, there is sufficient time and opportunity to discuss care-related issues among nurses | 1.00 | 10 |
| 20 | My job requires a high level of professional competence | .64 | ***Removed*** | | |
| 21 | Family-related anxieties and concerns interfere with my work activities | .82 | ***Removed*** | | |
| 22 | I feel so tired and stressed after work that it is difficult to fulfill family responsibilities | .91 | I feel so tired and stressed after work that it is difficult to fulfill family responsibilities | .91 | 5 |
| 23 | The nurse manager involves nurses in work-related decisions | .82 | In my unit, the nurse manager involves nurses in work-related decisions | 1.00 | 17 |
| 24 | Colleagues listen to each other and try to accommodate each other's needs | .73 | In my unit, colleagues listen to each other and try to accommodate each other's needs | .91 | 11 |
| 25 | There is sufficient healthcare staff (physicians, nurses, support staff) to carry out the work | .91 | In my unit, there is sufficient staff to ensure the delivery of clinical activities | .91 | 1 |
| 26 | I have the opportunity to acquire specific skills and competencies | .64 | ***Removed*** | | |
| 27 | I am required to work very fast | .91 | In my unit, I am required to work very fast | .91 | 27 |
| 28 | I deal with demanding users | .82 | During my work, I deal with demanding patients and family members | .91 | 12 |
| 29 | Work commitments force me to change my family plans | .73 | Work commitments force me to change my family plans | .82 | 7 |
| 30 | The behavior of my nurse manager is consistent with stated goals | .82 | In my unit, the behavior of the nurse manager is consistent with stated goals | .82 | 16 |
| 31 | I am required to work hard | .82 | In my unit, I am required to work hard | .82 | 26 |
| 32 | The number of nurses on duty is sufficient to ensure high-quality patient care | .73 | In my unit, there are enough nurses to ensure high-quality patient care | .91 | 3 |
| 33 | I deal with users who complain continuously despite my efforts to help them | .73 | During my work, I deal with patients who complain continuously despite my efforts to help them | .82 | 13 |
| 34 | There is not enough time to complete my tasks | .91 | In my unit, I do not have enough time to complete my tasks | .91 | 28 |
| 35 | Adequate support services allow sufficient time to be devoted to patients | .82 | In my unit, there are adequate support services to allow sufficient time to be devoted to patients | .91 | 4 |
| 36 | I can decide how to carry out my tasks | .91 | In my unit, I have the freedom to decide how to carry out my tasks | 1.00 | 20 |
| 37 | nurses and physicians deal with common work situations. | .73 | In my unit, nurses and physicians share many work activities. | .91 | 24 |
| 38 | The tasks I have to perform require knowledge and skills that I possess | .64 | ***Removed*** | | |

**Note. #items = number of items; I-CVI = item-level content validity index; Q-CVI = questionnaire-level content validity index. The Q-CVI was .78 in Round 1 and .91 in Round 2.**

| | | Factor Loadings | | | | | | | |
|---|---|---|---|---|---|---|---|---|---|
| | Comunalities | 1 | 2 | 3 | 4 | 5 | 6 | 7 | 8 |
| Items_1 | 0,764 | 0,121 | -0,027 | 0,016 | 0,133 | -0,042 | 0,018 | 0,802 | -0,133 |
| Items_2 | 0,474 | 0,048 | 0,095 | 0,03 | 0,147 | 0,039 | -0,092 | 0,121 | 0,527 |
| Items_3 | 0,882 | -0,02 | -0,006 | -0,043 | -0,03 | 0,047 | -0,028 | 0,944 | 0,013 |
| Items_4 | 0,554 | -0,013 | -0,11 | 0,016 | -0,066 | -0,087 | -0,031 | 0,611 | 0,198 |
| Items_5 | 0,684 | 0,004 | 0,118 | 0,075 | -0,045 | 0,71 | 0,131 | 0,019 | -0,014 |
| Items_6 | 0,763 | -0,038 | -0,101 | -0,05 | 0,009 | 0,893 | 0,039 | -0,002 | 0,002 |
| Items_7 | 0,621 | 0,038 | 0,014 | -0,003 | 0,009 | 0,801 | -0,07 | -0,029 | 0,032 |
| Items_8 | 0,438 | 0,141 | 0,142 | -0,178 | 0,038 | 0,031 | -0,117 | 0,01 | 0,45 |
| Items_9 | 0,483 | -0,053 | 0,101 | 0,032 | 0,064 | 0,029 | -0,041 | 0,048 | 0,667 |
| Items_10 | 0,537 | 0,082 | -0,243 | -0,041 | 0,047 | -0,059 | 0,084 | 0,243 | 0,448 |
| Items_11 | 0,616 | 0,133 | -0,146 | -0,069 | 0,047 | -0,084 | 0,102 | -0,112 | 0,685 |
| Items_12 | 0,603 | -0,017 | 0,07 | -0,112 | 0,021 | -0,035 | 0,769 | 0,013 | 0,04 |
| Items_13 | 0,774 | -0,015 | 0,071 | 0,022 | 0,059 | 0,036 | 0,834 | -0,001 | -0,06 |
| Items_14 | 0,49 | -0,016 | -0,005 | 0,123 | -0,042 | 0,08 | 0,624 | -0,035 | -0,018 |
| Items_15 | 0,521 | -0,023 | 0,055 | -0,023 | 0,718 | 0,04 | 0,028 | 0,015 | 0,008 |
| Items_16 | 0,857 | 0,008 | -0,038 | 0,068 | 0,94 | -0,028 | -0,038 | -0,01 | 0 |
| Items_17 | 0,716 | 0,033 | -0,102 | -0,084 | 0,749 | -0,038 | 0,057 | -0,026 | 0,07 |
| Items_18 | 0,548 | 0,127 | -0,008 | -0,654 | 0,034 | -0,028 | -0,011 | 0,029 | -0,021 |
| Items_19 | 0,651 | -0,044 | -0,049 | -0,844 | -0,035 | 0,085 | 0,073 | -0,027 | -0,005 |
| Items_20 | 0,799 | 0,018 | -0,049 | -0,869 | 0,019 | -0,01 | -0,022 | -0,032 | 0,002 |
| Items_21 | 0,654 | -0,026 | 0,082 | -0,761 | 0,054 | -0,085 | -0,07 | 0,056 | 0,027 |
| Items_22 | 0,864 | 0,939 | -0,053 | 0,009 | -0,008 | -0,037 | -0,011 | -0,071 | 0,03 |
| Items_23 | 0,815 | 0,87 | -0,018 | 0,006 | -0,008 | -0,015 | 0,026 | 0,025 | 0,071 |
| Items_24 | 0,577 | 0,654 | 0,115 | -0,072 | 0,066 | 0,067 | -0,078 | 0,133 | -0,067 |
| Items_25 | 0,622 | 0,081 | 0,743 | -0,008 | -0,029 | -0,045 | 0,136 | -0,01 | 0,079 |
| Items_26 | 0,759 | 0,066 | 0,834 | 0,017 | -0,011 | 0,041 | 0,045 | -0,009 | 0,014 |
| Items_27 | 0,65 | -0,092 | 0,799 | -0,011 | 0,007 | -0,013 | -0,017 | -0,042 | 0,015 |
| Items_28 | 0,545 | -0,027 | 0,608 | 0,086 | -0,043 | 0,096 | 0,039 | -0,089 | -0,056 |
| Extraction method: ML; Rotation: Oblimin with Kaiser normalizzation;Convergence for rotation in 9 interaction | | | | | | | | | |
